# Impact of trastuzumab emtansine (T-DM1) on spleen volume in patients with HER2-positive metastatic breast cancer

**DOI:** 10.1093/jjco/hyae141

**Published:** 2024-10-10

**Authors:** Arif Akyildiz, Rashad Ismayilov, Najmaddin Abdurrahimli, Aylin Ormanci, Deniz Can Guven, Murat Tuncel, Mehmet Ruhi Onur, Sercan Aksoy

**Affiliations:** Department of Medical Oncology, Hacettepe University Medical School, Ankara, Turkey; Department of Internal Medicine, Hacettepe University Medical School, Ankara, Turkey; Department of Radiology, Hacettepe University Medical School, Ankara, Turkey; Department of Nuclear Medicine, Hacettepe University Medical School, Ankara, Turkey; Department of Medical Oncology, Hacettepe University Medical School, Ankara, Turkey; Department of Nuclear Medicine, Hacettepe University Medical School, Ankara, Turkey; Department of Radiology, Hacettepe University Medical School, Ankara, Turkey; Department of Medical Oncology, Hacettepe University Medical School, Ankara, Turkey

**Keywords:** breast cancer, T-DM1, splenomegaly

## Abstract

**Background:**

Trastuzumab emtansine (T-DM1) is a novel therapy for human epidermal growth factor receptor 2 (HER2)-positive metastatic breast cancer, combining the targeted action of trastuzumab with the cytotoxic effects of emtansine. Although T-DM1 has demonstrated greater efficacy and safety compared to traditional therapies, concerns about hepatotoxicity and spleen-related complications have arisen.

**Methods:**

We conducted a retrospective study of 64 HER2-positive metastatic breast cancer patients treated with T-DM1 at our institution. Patients underwent computed tomography or magnetic resonance imaging at baseline and during treatment cycles. Spleen volume, portal vein diameter, and laboratory values were compared between baseline and 12 months after T-DM1 treatment.

**Results:**

Median spleen volume significantly increased from 201 cm^3^ (IQR, 157–275) at baseline to 291 cm^3^ (IQR, 215–420) after 12 months of T-DM1 treatment (*P* < 0.001). Spleen enlargement was observed in 87.5% of patients, while no significant alteration was detected in portal vein diameter. The change in spleen volume was positively correlated with changes in serum globulin levels, liver enzymes, and bilirubin levels, but did not impact survival outcomes.

**Conclusions:**

T-DM1 therapy in HER2-positive metastatic breast cancer leads to significant spleen enlargement and systemic biochemical changes. Future studies should focus on elucidating the long-term implications of these findings and optimizing monitoring strategies for spleen-related complications.

## Teaser

Trastuzumab emtansine (T-DM1) significantly increased spleen volume in human epidermal growth factor receptor 2 -positive metastatic breast cancer patients. Despite biochemical changes, no impact on survival was observed, warranting further investigation into long-term spleen-related complications.

## Introduction

Trastuzumab emtansine (T-DM1) represents a paradigm shift in cancer therapy [1]. This innovative treatment combines trastuzumab, a monoclonal antibody directed against the human epidermal growth factor receptor 2 (HER2), with the potent cytotoxicity of emtansine, an antimicrotubule agent. T-DM1 delivers emtansine directly into HER2-expressing cells through the trastuzumab component [2,3]. This mechanism mitigates the collateral damage often associated with conventional chemotherapy, offering renewed optimism to patients with HER2-positive metastatic breast cancer [4,5].

T-DM1’s journey from research to clinical application has been marked by pivotal clinical trials, culminating in its approval for patients who have previously undergone treatment with trastuzumab-based regimens [6,7]. The first-line treatment for newly diagnosed HER2-positive metastatic breast cancer is a combination of pertuzumab, trastuzumab, and a taxane. If the disease progresses, T-DM1 is the recommended second-line therapy, as shown by the EMILIA trial [7], which reported a median progression-free survival (PFS) of 9.6 months and a median overall survival (OS) of 30.9 months. Comparative analyses have highlighted T-DM1’s superiority over therapies like lapatinib and capecitabine, showcasing enhanced effectiveness and a favorable safety profile [8,9]. Phase III trials have further reinforced this safety profile, revealing a reduced incidence of grade 3 or higher adverse events with T-DM1 compared to its counterparts [9–11]. Nevertheless, concerns have emerged regarding potential hepatotoxicity and spleen-related complications of T-DM1. Case reports and clinical observations have indicated a possible link between T-DM1 therapy and spleen enlargement, accompanied by manifestations of portal hypertension such as spontaneous portal systemic shunt and hepatic nodular regenerative hyperplasia [12,13]. It is established that thrombocytopenia, occurring in 32.2% of patients treated with T-DM1, is the most common side effect, but its exact mechanism is not fully understood. Hypersplenism has been suggested as a possible contributing factor [14,15]. Recognizing these potential adverse events, our study explores the correlation between T-DM1 exposure and alterations in spleen volume.

## Patients and methods

### Patient selection

This retrospective study included 64 HER2-positive metastatic breast cancer patients who received T-DM1 as a second-line therapy following trastuzumab, with or without pertuzumab, at our institution between January 2017 and February 2024. Inclusion criteria required patients to have undergone computed tomography (CT) scan, magnetic resonance imaging (MRI), or 18F-fluorodeoxyglucose positron emission tomography/computed tomography (18F-FDG PET/CT) within 3 months prior to initiating T-DM1 and at least once during subsequent therapy cycles. Patients with a history of splenectomy were excluded. During the study period, T-DM1 was administered intravenously at a dose of 3.6 mg/kg every 21 days, with treatment continuing until disease progression or the occurrence of unacceptable toxicity. Approval for the study was obtained from the institutional ethics committee (Date: 20.02.2024, Decision number: 2024/04-19).

### Data collection

Study data were obtained from the hospital’s electronic database. At the initiation of T-DM1 treatment, patient age and Eastern Cooperative Oncology Group—performance status (ECOG-PS), comorbid diseases, hormone status, distant metastasis site, and previous treatments were recorded. Laboratory data included complete blood count, albumin, globulin, alanine aminotransferase (ALT), aspartate aminotransferase (AST), alkaline phosphatase (ALP), gamma-glutamyl transferase (GGT), lactate dehydrogenase (LDH), and bilirubin levels. The maximum cranio-caudal distance (L, cm), longest dimension in the axial plane (D, cm), and greatest thickness (T, cm) of the spleen as well as portal vein diameter (mm) were measured by CT or MRI. Spleen volume (cm^3^) was calculated with the formula ‘30 + 0.58 × L × D × T’. CT images of the 18F-FDG PET/CT acquisitions were evaluated on the Simplict90Y Personalized Dosimetry programm. Spleen contours were drawn manually and spleen volume was calculated. Although all patients had imaging at the 12th month of treatment, images at 3, 6, or 9 months were not available in all cases. Therefore, based on images before T-DM1 and at the 12th month of treatment, the percentage changes in spleen volume were analysed. Patients who received T-DM1 for less than 12 months were treated with chemotherapy or lapatinib for the remaining follow-up.

### Statistical analysis

All analyses were performed with the Statistical Package for Social Sciences, version 27 (IBM Inc., Armonk, NY). Descriptive statistics were presented as frequency (percent), mean ± standard deviation (SD), or median (interquartile range, IQR). Continuous variables were investigated with visual and analytical methods to determine the normal distribution. The Wilcoxon signed-rank test was utilized to compare two non-parametric dependent samples, while the Mann–Whitney U test was employed to compare two independent groups. The strength of the link between two continuous variables, at least one of which was not normally distributed, was demonstrated by Spearman's rho correlation coefficient. Survival estimates were calculated with the Kaplan–Meier method. Univariate survival analyses were conducted using the Cox regression method to obtain hazard ratios (HRs) with 95% confidence intervals (CIs). A 5% type-I error level was used to infer statistical significance.

## Results

The mean age of the 64 women included in the study was 56 ± 12.6 years. Forty-one (64.1%) patients exhibited at least one comorbidity, with diabetes (28.1%) and hypertension (25%) being the most frequently observed. No patient had cirrhosis, portal vein thrombosis, benign or malignant hematological disease, or other disorders characterized primarily by splenomegaly. Only 2 (3.1%) patients were chronic hepatitis B carriers. The baseline characteristics of the patients are detailed in Table 1.

**Table 1:** Patient characteristics

Characteristics	Frequency (%), *n = 64*
Age, mean ± SD, y	56 ± 12.6
Comorbidities	41 (64.1)
Diabetes mellitus	18 (28.1)
Hypertension	16 (25.0)
Hyperlipidemia	8 (12.5)
Hypothyroidism	7 (10.9)
Coronary artery disease	3 (4.7)
Others	14 (21.9)
ECOG-PS	
0	10 (15.6)
1	47 (73.4)
2	7 (10.9)
Hormone status	
Positive	30 (46.9)
Negative	34 (53.1)
Metastasis site	
Brain	30 (46.9)
Liver	23 (35.9)
Other	19 (29.7)
First-line anti-HER2 therapy	
Trastuzumab only	44 (68.8)
Trastuzumab plus pertuzumab	20 (31.2)

ECOG-PS: Eastern Cooperative Oncology Group—performance status, HER2: human epidermal growth factor receptor 2, SD: Standard deviation.

**Figure 1:**
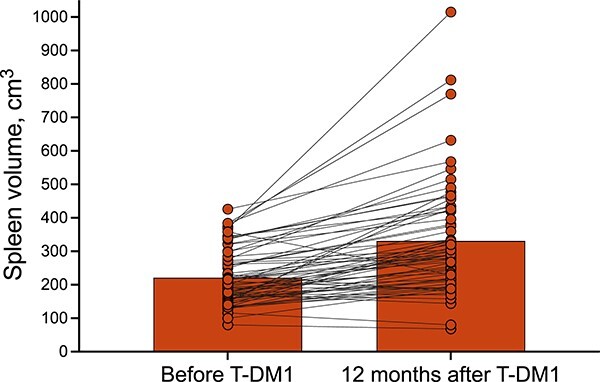
Spleen volumes before and 12 months after initiation of T-DM.

**Figure 2:**
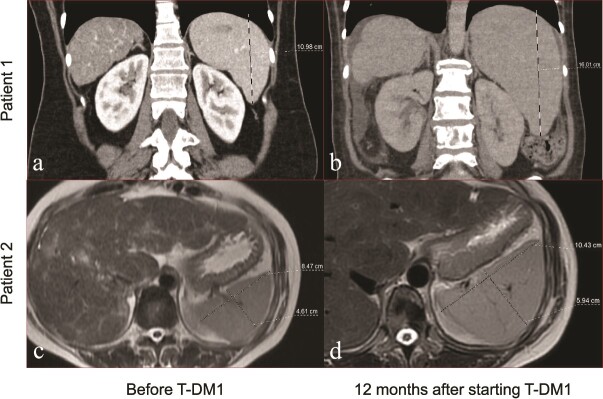
Two illustrative cases of splenomegaly: In patient 1, computed tomography reveals a 3.2-fold increase in spleen volume 12 months post-T-DM1 treatment (b) compared to baseline (a). In patient 2, magnetic resonance imaging demonstrates a 2-fold increase in spleen volume 12 months after the initiation of T-DM1 treatment (d) compared to baseline (c).

**Figure 3:**
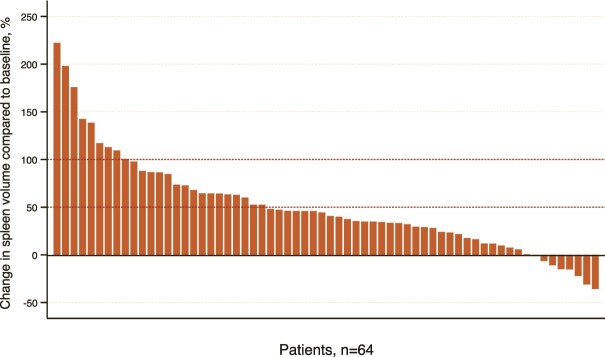
The waterfall graph illustrates the percentage change in spleen volume compared to baseline after 12 months of T-DM1 treatment.

The median spleen volume was measured as 203 cm^3^ (IQR, 171–259) before first-line therapy and 201 cm^3^ (IQR, 157–275) before T-DM1 treatment (*P* = 0.529). Twelve months after T-DM1, it showed a significant increase compared to pre-T-DM1 and reached 291 cm^3^ (IQR, 215–420) (*P* < 0.001, Figs 1 and 2). However, the median portal vein diameter before and 12 months after treatment was statistically similar [13 mm (IQR, 11–13) and 12 mm (IQR, 12–13), respectively, *P* = 0.195]. Spleen volume increased in 56 (87.5%) patients one year after starting T-DM1. Twenty-five (39.1%) patients exhibited a spleen volume increase of over 50%, while nine (14.1%) patients experienced an increase exceeding 100% (Fig. 3). The median percent change in spleen volume was 42.6% (IQR, 18.6–71.6) and showed no differentiation based on age (<60 vs. ≥60 years, *P* = 0.563), presence of comorbidities (*P* = 0.458), ECOG-PS score (0 vs. 1–2, *P* = 0.229), presence of liver metastases (*P* = 0.124), or prior dual anti-HER2 therapy (*P* = 0.143).

Among patients who received T-DM1 for less than 12 months, three patients exhibited an increase in spleen volume at 3 months (from 193 to 216 , 219 to 334 , and 336 to 490 cm^3^, respectively), while 15 patients showed an increase at 6 months (median 172 cm^3^ [IQR, 150–284] to 290 cm^3^ [IQR, 213–396]; *P* = 0.002). In these patients, spleen volume did not show significant change up to the 12th month (3rd or 6th month vs. 12th month, *P* = 0.176).

We conducted a comparative analysis of laboratory values before and 12 months after initiation of T-DM1. In the 12th month of treatment, there was a notable decline in median white blood cell (*P* < 0.001), neutrophil (*P* = 0.002), lymphocyte (*P* = 0.002), monocyte (*P* = 0.043), and platelet counts (*P* < 0.001) as well as a reduction in albumin levels (*P* < 0.001) compared to baseline levels at the initiation of treatment. Conversely, the temporal comparison revealed a significant increase in globulin (*P* < 0.001), ALT (*P* < 0.001), AST (*P* < 0.001), ALP (*P* < 0.001), GGT (*P* < 0.001), total bilirubin (*P* < 0.001), indirect bilirubin (*P* < 0.001), and direct bilirubin (*P* < 0.001) levels (Table 2). There was a positive correlation between spleen volume change and globulin (r = 0.436, *P* < 0.001), AST (r = 0.268, *P* = 0.033), ALP (r = 0.301, *P* = 0.016), total bilirubin (r = 0.267, *P* = 0.033) and direct bilirubin (r = 0.266, *P* = 0.034) changes before and 12 months after the onset of T-DM1. Alterations in other laboratory findings showed no significant correlation with changes in spleen volume (Table 3). Cirrhosis developed in two patients by the 12th month of T-DM1 treatment. In one of these patients, grade 2 varices were detected on upper endoscopy in the second year of treatment. However, no patient exhibited clinical signs of decompensated cirrhosis, such as jaundice, ascites, variceal bleeding, or encephalopathy.

**Table 2:** Comparison of radiological and laboratory parameters before and 12 months after the onset of T-DM1

Parameters, median (IQR)	Before T-DM1	At 12^th^ month of T-DM1	*P* value^a^^,^^b^
Spleen volume, cm^3^	201 (157–275)	291 (215–420)	**<0.001**
Portal vein diameter, mm	13 (11–13)	12 (12–13)	0.195
Hemoglobin, g/L	12.7 (11.9–13.4)	12.3 (11.0–13.4)	0.081
WBC, x10^3^/mcL	5.8 (4.6–7.7)	4.6 (3.9–6.0)	**<0.001**
Neutrophil, ×10^3^/mcL	3.5 (2.8–4.6)	2.8 (2.1–4.0)	**0.002**
Lymphocyte, ×10^3^/mcL	1.5 (1.0–1.9)	1.3 (1.0–1.5)	**0.002**
Monocyte, ×10^3^/mcL	0.5 (0.4–0.7)	0.4 (0.4–0.6)	**0.043**
Platelet, ×10^3^/mcL	222 (187–275)	137 (103–188)	**<0.001**
Albumin, g/dl	4.1 (4.0–4.3)	4.0 (3.6–4.2)	**<0.001**
Globulin, g/dl	3.1 (2.8–3.4)	3.4 (3.0–3.9)	**<0.001**
ALT, U/L	20 (15–31)	29 (21–42)	**<0.001**
AST, U/L	25 (21–34)	40 (30–56)	**<0.001**
ALP, U/L	92 (73–115)	121 (85–182)	**<0.001**
GGT, U/L	27 (18–50)	54 (26–157)	**<0.001**
LDH, U/L	223 (194–275)	244 (200–313)	0.072
Total bilirubin, mg/dl	0.45 (0.35–0.56)	0.89 (0.60–1.15)	**<0.001**
Indirect bilirubin, mg/dl	0.36 (0.28–0.46)	0.65 (0.46–0.85)	**<0.001**
Direct bilirubin, mg/dl	0.09 (0.07–0.11)	0.18 (0.12–0.26)	**<0.001**

^a^Wilcoxon signed-rank test.

^b^Bold text indicates statistical significance at *P* < 0.05 (2-sided).

ALP: alkaline phosphatase, ALT: alanine aminotransferase, AST: aspartate aminotransferase, GGT: gamma-glutamyl transferase, IQR: interquartile range, LDH: lactate dehydrogenase, T-DM1: trastuzumab emtansine, WBC: white blood cell.

**Table 3:** Correlation analysis between the change in spleen volume and the changes in laboratory findings before and 12 months after the T-DM1 initiation

Changes in laboratory parameters	Change in spleen volume	
	Correlation coefficient^a^	*P* value^b^
Hemoglobin	− 0.001	0.991
WBC	−0.040	0.757
Neutrophil	0.048	0.705
Lymphocyte	− 0.031	0.811
Monocyte	0.042	0.743
Platelet	−0.056	0.659
Albumin	−0.077	0.543
Globulin	0.436	**<0.001**
ALT	0.062	0.627
AST	0.268	**0.033**
ALP	0.301	**0.016**
GGT	0.210	0.096
LDH	0.098	0.443
Total bilirubin	0.267	**0.033**
Indirect bilirubin	0.202	0.110
Direct bilirubin	0.266	**0.034**

^a^Spearman’s rho.

^b^Bold text indicates statistical significance at *P* < 0.05 (2-tailed).

ALP: alkaline phosphatase, ALT: alanine aminotransferase, AST: aspartate aminotransferase, GGT: gamma-glutamyl transferase, LDH: lactate dehydrogenase, T-DM1: trastuzumab emtansine, WBC: white blood cell.

During the median follow-up period of 24.5 months (IQR, 14.4–39.1) after the initiation of T-DM1, 49 (76.6%) patients progressed and 35 (54.7%) patients died. Median PFS and OS estimates were 9.9 months (95% CI: 6.2–13.6) and 37 months (95% CI: 25.9–48.1), respectively. Univariate analyses showed that the percentage change in spleen volume over one year did not affect PFS (HR: 0.998, 95% CI: 0.992–1.004, *P* = 0.452) or OS (HR: 1.001, 95% CI: 0.995–1.007, *P* = 0.738).

## Discussion

In this study, we showed a correlation between T-DM1 treatment and splenomegaly in patients with HER2-positive metastatic breast cancer. Our findings reveal a significant increase in spleen volume following T-DM1 initiation, with a substantial proportion of patients exhibiting splenomegaly. Notably, the incidence of spleen enlargement prompts consideration of potential mechanisms underlying this phenomenon.

The absence of differentiation based on demographic and clinical parameters highlights the indiscriminate nature of this effect. Although the pathophysiological mechanisms leading to portal hypertension associated with T-DM1 are not fully understood. Nonetheless, the literature has documented cases of hepatic nodular regenerative hyperplasia and portal hypertension following T-DM1 administration [12,16]. T-DM1 is known to undergo metabolism within the reticuloendothelial system, suggesting the potential for T-DM1 metabolism to directly or indirectly induce reticuloendothelial hyperplasia [17]. Nodular regenerative hyperplasia represents a porto-sinusoidal vascular disorder resulting in non-cirrhotic portal hypertension. Animal studies have provided microscopic evidence, including hypertrophy and hyperplasia of splenic reticuloendothelial cells, along with lymphoid depletion of follicular centers in the spleen and thymus. Similarly, rat experiments have revealed analogous findings in response to T-DM1 exposure, leading to an increase in spleen weights [18]. Nonetheless, due to limited pathological evidence, further investigation is warranted to ascertain whether nodular regenerative hyperplasia contributes to T-DM1-associated portal hypertension.

Clinical data on the relationship of T-DM1 treatment with splenic enlargement is limited. Kosmin M et al [19] evaluated 12 patients with metastatic breast cancer with whole-body MRI before and during T-DM1 therapy. They observed an increase in spleen volume in 92% of patients, which correlated with treatment duration. Similar to our study, no significant change in portal vein diameter was reported. In another study, patients with HER2-positive metastatic breast cancer receiving T-DM1 were compared to those receiving lapatinib and capecitabine [13]. The T-DM1 cohort exhibited a significantly higher percentage of spleen enlargement at the 33^rd^ treatment cycle (104% ± 5 vs −1% ± 6, *P* < 0.001). Consistent with these findings, we found a median increase of 42.6% in spleen volume during the first year of T-DM1 treatment in our cohort. Our study also revealed concomitant changes in laboratory parameters, reflecting systemic effects induced by T-DM1 therapy. The notable decline in hematological indices, particularly platelet counts, underscores the hematologic toxicity associated with T-DM1 administration. Conversely, the observed elevation in liver enzymes and bilirubin levels suggests hepatocellular injury, corroborating evidence of hepatotoxicity associated with T-DM1.

Despite these notable observations, the clinical implications of spleen enlargement and portal hypertension in the context of T-DM1 therapy remain uncertain. Our study, while shedding light on these phenomena, does not demonstrate a significant impact on PFS or OS. However, the implications of prolonged exposure to T-DM1 warrant further investigation, particularly in elucidating the long-term sequelae of spleen-related complications. While our study primarily examined radiological and laboratory findings associated with portal hypertension, it is crucial that patients also undergo surveillance for additional manifestations, including gastroesophageal varices and spider angiomas. Although there were no instances of gastrointestinal bleeding within our cohort, we recommend monitoring for varices in patients receiving T-DM1 therapy. Literature reports of patients developing T-DM1-associated varices underscore the importance of such vigilance [16,20].

Our study had several limitations, primarily its retrospective design. Secondly, the timing and frequency of radiological examinations varied among patients, as these were determined based on clinical judgment. Nonetheless, each patient had sufficient follow-up time to assess spleen enlargement. Thirdly, patients in our cohort did not undergo endoscopic examination, likely due to an underrecognition of T-DM1-associated portal hypertension. The limited use of endoscopic monitoring and reliance solely on radiological criteria may have led to the potential oversight of additional signs of portal hypertension.

## Conclusion

In conclusion, our study contributes to the evolving discourse surrounding T-DM1 therapy, emphasizing the importance of monitoring spleen volume changes and potential indicators of portal hypertension. Ongoing surveillance and research are imperative to unravel the complex interplay between T-DM1 exposure, splenic dynamics, and hepatobiliary function.

## Compliance with Ethical Standards

All procedures performed in the study involving human participants were in accordance with the 1964 Helsinki declaration and its later amendments. The study was approved by Hacettepe University Ethics Board (Date: 20 Feb 2024, Decision no: 2024/04–19).

## Data Availability

All data that support the findings of this study are available within the article and from the corresponding author.
