# Correction to: Impact of trastuzumab emtansine (T-DM1) on spleen volume in patients with HER2-positive metastatic breast cancer

**DOI:** 10.1093/jjco/hyae166

**Published:** 2024-11-12

This is a correction to: Arif Akyildiz, Rashad Ismayilov, Najmaddin Abdurrahimli, Aylin Ormanci, Deniz Can Guven, Murat Tuncel, Mehmet Ruhi Onur, Sercan Aksoy, Impact of trastuzumab emtansine (T-DM1) on spleen volume in patients with HER2-positive metastatic breast cancer, *Japanese Journal of Clinical Oncology*, 2024, hyae141, https://doi.org/10.1093/jjco/hyae141

In the originally published version of this paper, there was a typographical error in the name of author Sercan Aksoy.

This has been corrected.

